# Whole-Exome Sequencing Identifies a Novel Variant (c.1538T > C) of *TNNI3K* in Arrhythmogenic Right Ventricular Cardiomyopathy

**DOI:** 10.3389/fcvm.2022.843837

**Published:** 2022-02-22

**Authors:** Ting Xie, Yifeng Yang, Ke Gong, Yong Luo, Hui Guo, Ruilin Liu, Lei Wang, Zhiping Tan, Jinwen Luo, Li Xie

**Affiliations:** ^1^Department of Cardiovascular Surgery, The Second Xiangya Hospital of Central South University, Central South University, Changsha, China; ^2^The Clinical Center for Gene Diagnosis and Therapy, The Second Xiangya Hospital of Central South University, Central South University, Changsha, China; ^3^Department of Cardio-Thoracic Surgery, Hunan Children's Hospital, Changsha, China

**Keywords:** whole-exome sequencing, arrhythmic right ventricular cardiomyopathy, Gene Mutation, *TNNI3K*, *RYR2*

## Abstract

**Backgrounds:**

Arrhythmic right ventricular cardiomyopathy (ARVC) is a cardiomyopathy with a genetic predisposition that can lead to a sudden cardiac death and heart failure. According to the 2010 Task Force Criteria, genetic diagnosis is one of the most important methods, but, so far, only a few genes related to ARVC have been identified.

**Methods:**

In this study, the pathogenic gene of a patient with ARVC was examined using whole-exome sequencing. The plasmids of *TNNI3K* were constructed, and the effects of the *TNNI3K* variant was investigated by a real-time polymerase chain reaction (PCR) and western blot.

**Results:**

A novel variant (c.1538T > C) of *TNNI3K* was identified, with phenotypes of dominant right ventricular (RV) disease preliminarily fulfilling the diagnosis of ARVC. A comprehensive assessment revealed that the variant was pathogenic. We found that this variant would lead to a decrease in the level of *TNNI3K* mRNA and protein, as well as a decrease in the expression of the *RYR2* gene, which further proves that *TNNI3K* plays an important role in cardiomyopathy and expands the spectrum of the *TNNI3K* variants.

**Conclusion:**

In this study, we reported a *TNNI3K* variant in ARVC for the first time, and the results not only contribute to the diagnosis of ARVC, but also provide a reference for genetic counseling and promote the understanding of the genetic mechanism of cardiomyopathy.

## Introduction

Arrhythmogenic right ventricular cardiomyopathy (ARVC) is an inherited cardiomyopathy characterized by ventricular arrhythmias and sudden death (1, 2). Fibrofatty replacement and right ventricle (RV) dilation are considered as the pathological features of ARVC (3). Clinical symptoms include palpitations, heart failure, dilatation and aneurysms of the right ventricle, and sudden death (3). There are many clinical methods for evaluating the right ventricular abnormalities and for differential diagnosis, e.g., RV/LV ratio and cardiac magnetic resonance (4). The ARVC can be inherited in many ways, but the autosomal-dominant inheritance is the most common (2). To date, some genes have been associated with the ARVC phenotype including desmosomal genes (*PKP2, DSG2, DSP, DSC2*, and *JUP*) and non-desmosomal genes (*LMNA, CTNNA3, CDH2, DES, TTN, PLN, RYR2, SNC5A*, and *TP63*) (1). The ARVC is primarily thought to be a lesion of the desmosome, or the cell-cell junctions at the intercalated disk. Pathogenic variants of five genes (*DSP, PKP2, DSG2, DSC2*, and *JUP*) are thought to play a significant role in the ARVC pathogenesis (5). Owing to the importance of identifying those at risk for sudden death, gene-finding efforts have continued.

The *TNNI3K* encodes the protein TNNI3-interacting kinase, which is located at the sarcomere Z disc (6). The *TNNI3K* protein includes a coiled-coil domain, ankyrin repeats, kinase domain, and serine-rich domain. Pathogenic variants are more concentrated in the kinase domain, including the variant we reported [Figure 2A, the domain of *TNNI3K* was downloaded from SMART (http://smart.embl.de/smart/show_motifs.pl)] (7–12). The *TNNI3K* expression greatly accelerates the cardiac dysfunction in cardiomyopathy mouse models (6). To date, no variant of *TNNI3K* has been detected in patients with ARVC. Here, we report a patient with ARVC who was screened for variants by whole-exome sequencing and found that the patient had a novel variant of *TNNI3K* (c.1538T>C).

## Materials and Methods

### Subjects

The study participant provided informed consent. The ARVC was diagnosed according to the 2010 Task Force Criteria (2). Blood sample was collected from the patient.

### DNA Extraction

Genomic DNA was extracted from the peripheral blood of patient. Genomic DNA was prepared using the DNeasy Blood & Tissue Kit (Qiagen, Valencia, CA, USA).

### Whole-Exome Sequencing

The main part of the whole exome sequencing (WES) was provided by the Berry Genomics Bioinformatics Institute (Beijing, China). Exomes were captured using Agilent SureSelect Human All Exon V6 kits, and the platform of high-throughput sequencing was performed on an Illumina HiSeq X-10. Basic bioinformatics analysis, including Reads, Mapping, Variant detection, Filtering, and Annotation, was also performed by the Berry Genomics Bioinformatics Institute.

### Variant Validation

The filtered variant was validated by Sanger sequencing. Primer pair were designed by Integrated DNA Technologies (IDT, https://sg.idtdna.com/pages), and sequences of the polymerase chain reaction (PCR) products were determined by Tsingke Biological Technology (Beijing, China).

### Cell Culture

The AC16 cardiomyocytes were cultured in Dulbecco's Modified Eagle Medium/F12 medium (Gibco, USA), supplemented with 10% fetal calf serum (Gibco), and 1% penicillin-streptomycin solution (100x; Solarbio), then incubated in an incubator containing 5% CO_2_ at 37°C.

### Cell Transfection

Wild-type *TNNI3K* CDS and L513P-*TNNI3K* missense variant plasmids, with HIS-tags in a pCMV3 vector, were designed. The AC16 cells were transfected with the *TNNI3K*-pCMV3-C-HIS (WT and c.1538T>C/p. L513P) using Lipofectamine^TM^ 3000 CD Transfection Reagent (Thermo Fisher Scientific), following the manufacturer's instructions.

### Real-Time PCR

Total RNA was extracted from AC16 cells transfected with WT and mutant plasmids using a GeneJET RNA Purification Kit (Thermo Fisher Scientific). The cDNA was synthesized from 1 μg of RNA using 4 × EZscript Reverse Transcription Mix II (with gDNA Remover; EZBioscience). A real-time PCR was carried out in a Fast 7500 Real-Time PCR System (Applied Biosystems) using the PowerUp™ SYBR™ Green Master Mix (ThermoFisher Scientific), and the 2(^−Δ*ΔCt*^) method was used to compare the mRNA expression levels between cells transfected with WT and mutant plasmids. The housekeeping gene, *GAPDH*, was used as the control. Each assay was performed in three independent tests. The primer sequences used for the qPCR are listed in Supplementary Table 1.

### Western Blot

For the western blot analysis, the harvested cells were homogenized in a lysis buffer on ice. The lysates (20 μg protein for each lane) were separated by 10% SDS-PAGE and transferred to 0.22 mm polyvinylidene fluoride (PVDF) membranes. Membranes were blocked with 5% non-fat milk for 2 h, and then incubated with primary antibodies overnight at 4°C. The secondary antibodies were then incubated with the membranes for 1 h. Western blots were visualized using an enhanced chemiluminescence. The antibodies against His-tag (cat. no. 66005, 1:1,000 dilution) and GAPDG (cat. no. 60004, 1:10,000 dilution) were purchased from Proteintech (USA).

### Multiple Sequence Alignment Analysis

The sequence of the *TNNI3K* protein for the different species was downloaded from the ensemble (https://asia.ensembl.org/index.html). A multiple sequence analysis was performed by ClustalX.

### Protein Structural Modeling

Homology modeling was performed to generate a 3D model of the kinase domain of *TNNI3K* using the SWISS-MODEL server (13).

### Statistical Analysis

Statistical analyses were performed using GraphPad Prism 8 software. All data in the present study were expressed as the mean ± SEM and were analyzed using a homogeneity of variance test and intergroup variance analysis. Statistical significance was set at *P* < 0.05. All the experiments in our research were repeated at least three times.

## Results

### Clinical Data

**Figure 1:**
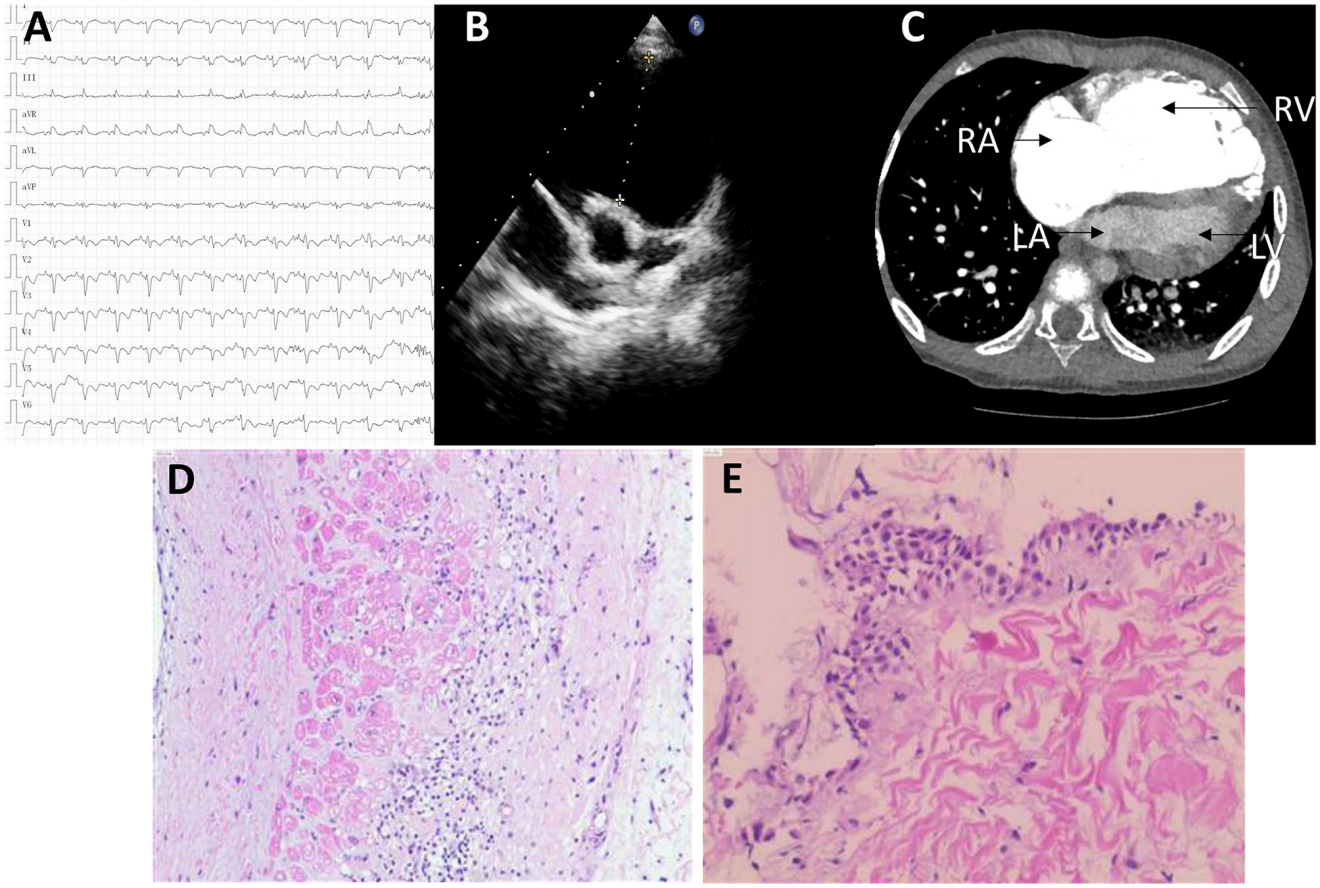
The clinical data. **(A)** The ECG showing a complete right bundle branch block with T- wave inversion V1–V6. **(B,C)** The B ultrasonic **(B)** and CT image **(C)** showing severe the right atrium (RA), right ventricle (RV), and right ventricle outflow tract (RVOT) dilatation. **(D,E)** The histopathological results of endocardial and myocardial tissue showing local muscle thinning and fibrous tissue hyperplasia edema. ECG, electrocardiogram; CT, computed tomography; RA, right atrium; RV, right ventricle; RVOT, outflow tract of right ventricle.

A 6-year-old boy was presented with a recurrent syncope. His ECG showed a complete right bundle branch block, with a T- wave inversion from V1 to V6 (Figure 1A). Echocardiogram demonstrated a pulmonary valve absence, severe dilatation of the right atrium (RA), RV, and RV outflow tract (RVOT) dilatation (RVOT 46 mm) (Figure 1B). The left ventricle (LV) showed a normal structure and function (LVEF, 77%; LVFS, 44%). Computed tomography (CT) further confirmed that the right heart was significantly enlarged (Figure 1C). The patient was diagnosed with pulmonary valve absence before surgery, and the pulmonary valve plasty + Glenn procedure was planned to be performed. However, during the surgery, the development of pulmonary valve was found to be normal; the RVOT is obviously dilated, the ventricular wall was thin, and the endocardium showed fibrous changes. Small pieces of endocardium and RV myocardium were removed and sent for examination. A pathological detection revealed a local muscle thinning and fibrous tissue hyperplasia edema (Figures 1D,E). To improve the right heart function, Glenn procedure was performed. According to these, the preliminary diagnosis is ARVC. The postoperative color doppler echocardiography indicated that the anastomosis of superior vena cava and right pulmonary artery had an unobstructed blood flow, while the right heart was still large.

### Genetic Analysis

**Figure 2:**
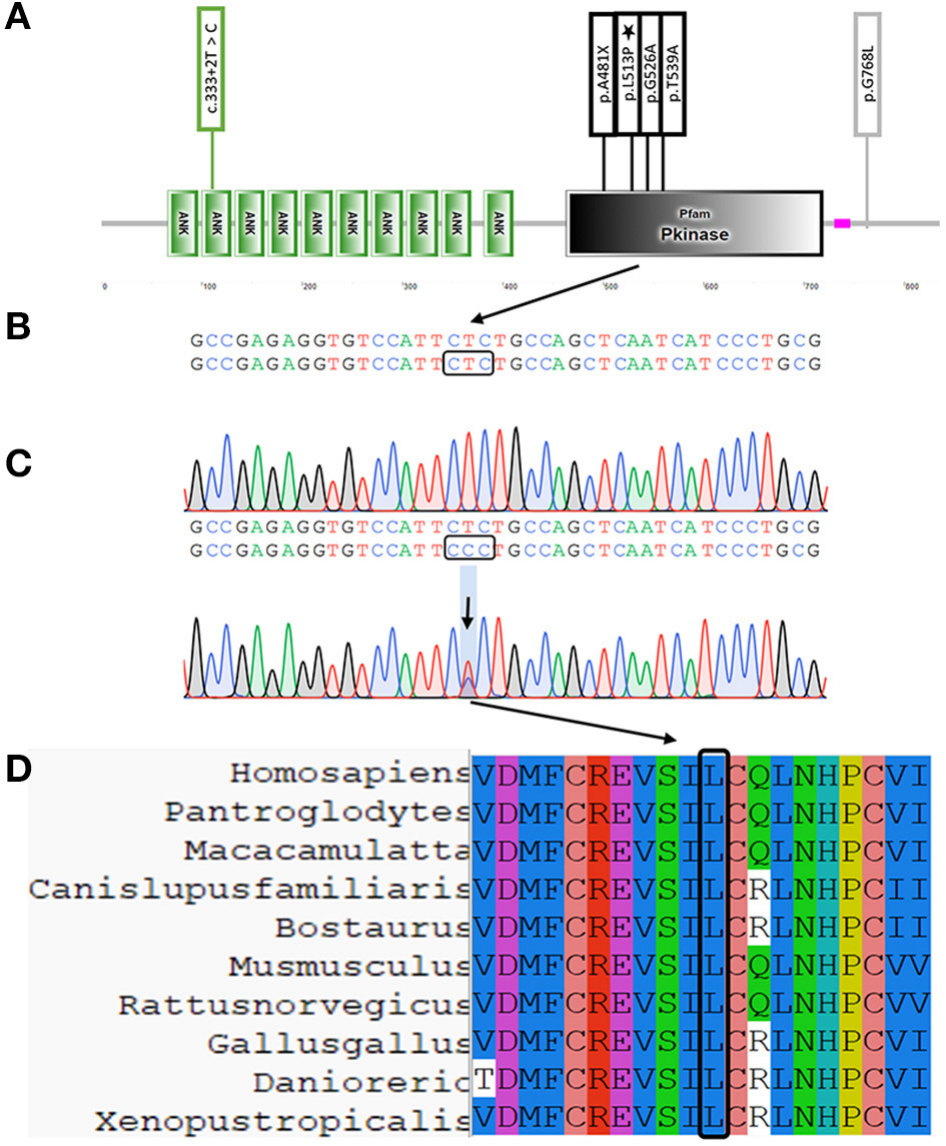
Location and conservation of *TNNI3K* L513P variant. **(A)** Schematic diagram of *TNNI3K* protein structure and variants' location. Asterisks represent a variant identified in this study **(B)** The protein topology is shown for *TNNI3K*, including 10 functional ankyrin repeat domains, a serine-rich domain (purple), and a single functional kinase domain where the heterozygous c.1538 T>C resides (black). **(C)** Sanger sequencing verified the variant. **(D)** Conservation of this residue.

**Figure 3:**
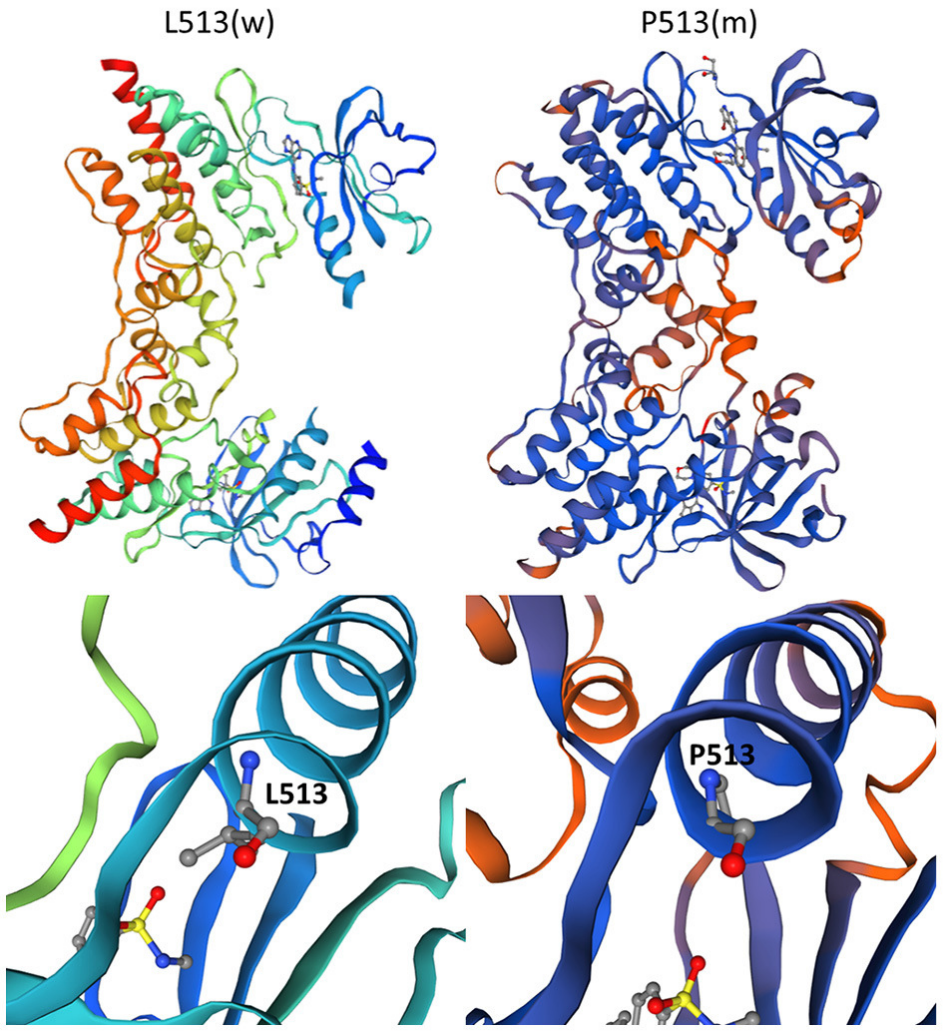
The 3D models of *TNNI3K*. The left panel depicts wild-type and the right panel shows mutant *TNNI3K*.

Whole-exome sequencing yielded 8.97 Gb of data with 99.9% coverage of the target regions and a depth of more than 50X. We then performed the data filtering as follows: (1) exclude the non-coding region and synonymous variants that have no impact on splicing [predicted by regSNP-intron (14), dbscSNV_ADA_SCOR, and dbscSNV_RF_SCORE < 0.6 (15)], and variants in 1,000 g, the Exome Aggregation Consortium (ExAC), and gnomAD databases; (2) rank genes using Polyphen-2, SIFT, MutationTaster, and CADD; and (3) sanger sequencing examines variants. Only a novel non-synonymous variant [NC_000001.10: g.74835140T > C, NM_015978.2: c.1538T > C, NP_057062.1: p. (Leu513Pro)] of *TNNI3K* was identified by Sanger sequencing, which resulted in the L513P substitution (Figure 2C). The protein topology is shown for *TNNI3K*, including 10 functional ankyrin repeat domains, a serine-rich domain (purple), and a single functional kinase domain where the heterozygous c.1538 T > C resides (black) (Figure 2B). The amino acid sequence of *TNNI3K* is highly conserved, with its amino acid identity to the human *TNNI3K* ortholog for both the kinase domain and the entire protein (Figure 2D). The wild-type (L513) and mutant (P513) have different space structures (Figure 3).

### Pathogenicity Analysis of the Novel *TNNI3K* Variant

**Figure 4:**
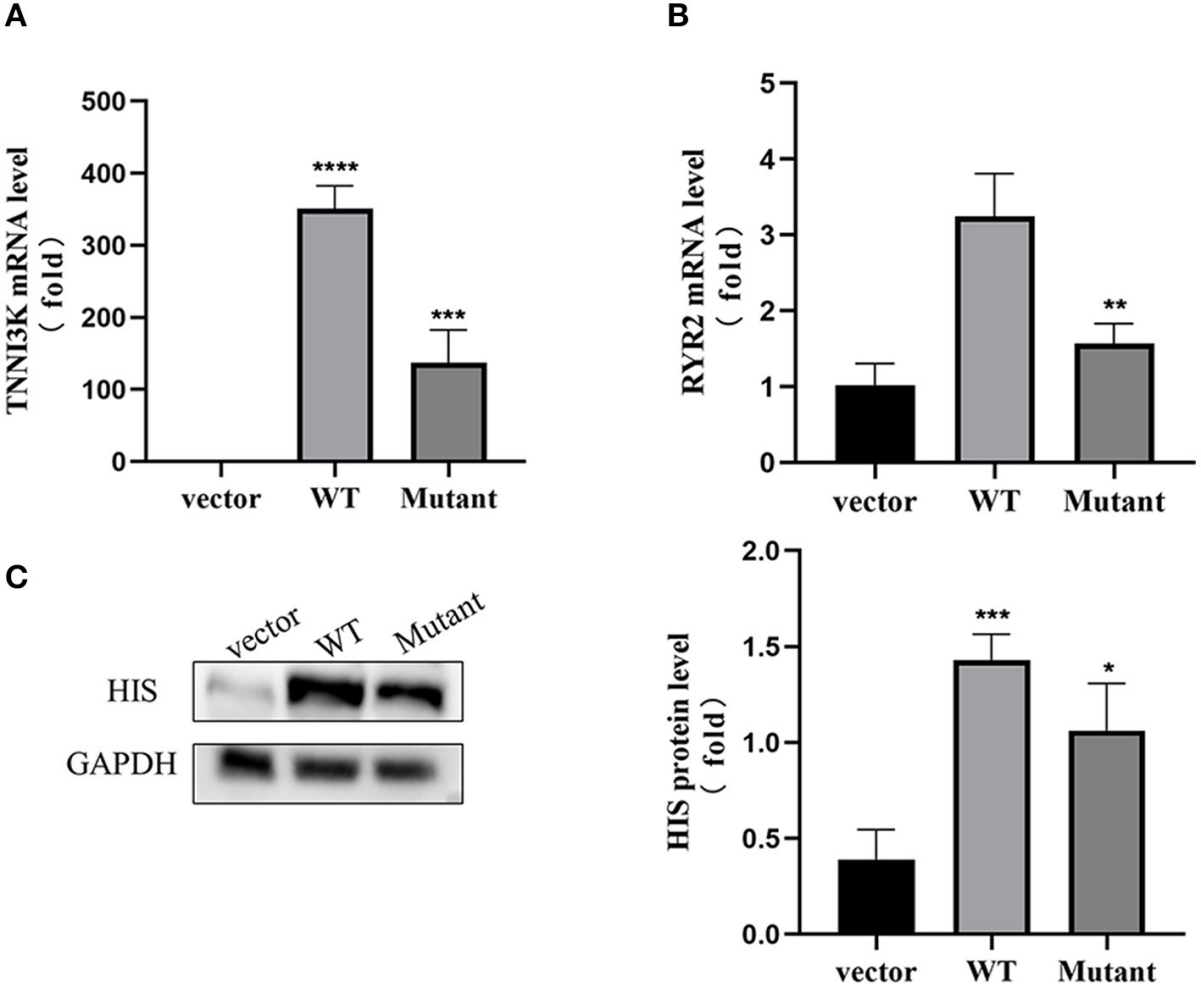
Pathogenicity evaluation of the missense variant of *TNNI3K* gene in AC16 cell model. **(A)** The mRNA level of *TNNI3K* in WT group was significantly increased compared to vector group, while the mRNA level was significantly decreased in mutant group compared with WT group (*n* = 4, ****p < 0.0001 vs. vector group, ***p < 0.001 vs. WT group). **(B)** The mRNA level of *RYR2* in mutant group was significantly decreased compared to WT group (*n* = 4, **p < 0.01 vs. WT group). **(C)** Western blots: DSG2 protein was increased in WT group compared with vector group, while the protein level was decreased in mutant group compared with WT group (*n* = 4, ***p < 0.001 vs. vector group, *p < 0.05 vs. WT group).

We used the American College of Medical Genetics (ACMG) assessment criteria to analyze the pathogenicity of the variant in detail and determined that the variant was likely pathogenic. The coding sequence of the human *TNNI3K* gene was cloned and the vectors carrying WT and mutant *TNNI3K* were constructed and were validated by Sanger sequencing (Supplementary Figure 1). Compared with pCMV3-C-HIS group, the mRNA level of *TNNI3K* in the WT group was significantly increased (*p* < 0.01). Meanwhile, the expression of *TNNI3K* gene was significantly decreased in the mutant group compared to that in the WT group (*p* < 0.01) (Figures 4A,B), similar to the mRNA of *RYR2*, suggesting that this variant would decrease the expression of *TNNI3K* and *RYR2* genes. Western blots showed that the expression of *TNNI3K* protein could be found (Figure 4C), and the protein of *TNNI3K* in the mutant group was lower than that in the WT group (*p* < 0.05), indicating that the variant not only affects the transcription level, but also leads to changes in the translation level.

## Discussion

In this study, we employed a whole exome sequencing (WES) to explore the genetic lesions in a Chinese child with an ARCV. A novel non-synonymous variant in *TNNI3K* was identified. Although *TNNI3K* is involved in dilated cardiomyopathy (DCM), its involvement in ARVC has not been reported. Four pathogenicity assessment tools were used to predict that the variant was pathogenic. Subsequent functional assessment demonstrated that the missense variant not only affected the expression of *TNNI3K*, but also the expression of *RYR2*, which has been verified to be related to ARVC (1). This implies that this variant is associated with ARVC pathogenesis. According to the Human Gene Mutation Database, no variants in *TNNI3K* have been reported in patients with ARVC. This is the first worldwide report of a *TNNI3K* variant in a human patient with ARVC worldwide. By constructing the *TNNI3K* vector, it was proved that the variant not only affected the expression of autogenic proteins, but subsequently, the expression of ARVC-related gene (*RYR2*) changed accordingly. The child was an orphan and parental DNA samples were not available, so, the new variant could not be verified by parental samples. This variant was not found in 1,000 g and the Exome Aggregation Consortium (ExAC), but only one heterozygous variant was found in gnomAD. According to these, we used an American College of Medical Genetics (ACMG) assessment criteria to analyze the pathogenicity of the variant in detail and to determine that the variant was likely pathogenic (PS3 + PM2 + PP3) (16).

Although the genetic basis of ARVC and dilated cardiomyopathy (DCM) is different, a similar pathological mechanism can be observed. The overlap between ARVC and DCM has been well-described (1, 17, 18). Patients with *DSP* mutations can be presented with typical ARVC, biventricular cardiomyopathy, or isolated LV, as well as DCM (19). Recent research have found that truncating variants in the gene-encoding filamin C (*FLNCtv*) are associated with arrhythmogenesis and DCM, with a reportedly high risk of ventricular arrhythmia (20). In addition, many genes have been identified in ARVC and are listed as pathogenic genes in DCM, such as *PKP2, JUP, LMNA, DES, PLN*, and *SCN5A* (1, 21). Notably, Theis et al. found a *TNNI3K* mutation in a familial syndrome of conduction system disease, atrial tachyarrhythmia, and DCM, which was shown to modulate cardiac conduction and myocardial function in mice (9), similar to other studies (7). The expression of mutant *TNNI3K* decreased, which is consistent with the results of known pathogenic mutations in DCM and cardiac conduction disease (CCD) (7, 9).

Although ARVC is considered a desmosome-related disease, several non-desmosomal genes are currently believed to be involved. Quarta et al. revealed that four of 108 patients with ARVC were found to carry (Lamin A/C) *LMNA* mutations without the desmosome mutations, and three of them had severe RV involvement and died at follow-up (22). Recently, Brun et al. identified two *FLNCtv* variants in 156 patients with ARVC (23). Similar to other studies, this study expanded the spectrum of ARVC non-desmosome disease genes for this disorder.

The *TNNI3K* has been linked to a broad spectrum of cardiac phenotypes in mice, such as accelerated cardiomyopathy (24) and cardiac conduction (25). Although the exact molecular mechanisms need to be further clarified, these findings in mice convincingly showed that the *TNNI3K* plays an important role in cardiac disease (24, 25). Recent studies demonstrated that non-sense *TNNI3K* mutation, associated with cardiac conduction disease (CCD) and harboring the mutation c.1441C > T, has implicated a loss-of-function pathogenic mechanism with an autosomal-dominant inheritance pattern (11). The *TNNI3K* has also a Residual Variation Intolerance Score of 27%, indicating that its tolerance of variation is <73% of that of other genes (26). The cardiac-specific expression of *TNNI3K* and its established role in murine cardiac physiology have prompted a great interest in the modulation of *TNNI3K* expression as a potential therapeutic strategy for heart disease (27).

Previous studies have shown that *RYR2* mutations are associated with ARVC (28), and that *RYR2* is downregulated in ARVC (29), which is consistent with the results of this study, and affects the calcium regulation (30). Cerrone et al. revealed that the expression of *RYR2* was decreased in *PKP2* knockout mice, but there was no difference in its individual functions. Although calcium release is reduced and the sarcoplasmic reticulum is overloaded with calcium, the diastolic [Ca^2+^]i increased. Moreover, the mathematical simulations, including the *RYR2* decline, were manifested by an increased calcium transient amplitude and an enhanced excitation-contraction (e-c) coupling gain (30). This study has some limitations. It lacks a clear pedigree genetic map, but we carried out functional verification of gene variant, although it was not comprehensive.

In conclusion, a novel variant (c.1538T > C) of *TNNI3K* was identified in a Chinese patient with ARVC. Functional experiments further confirmed that this variant alters the expression of the ARVC-associated gene (*RYR2*). More importantly, *TNNI3K* has also not been previously reported in ARVC. Our study expands the spectrum of *TNNI3K* variants and may contribute to the genetic diagnosis and counseling for ARVC. The significant role of *TNNI3K* in heart development needs to be further confirmed.

## Data Availability Statement

The datasets presented in this study can be found in online repositories. The names of the repository/repositories and accession number(s) can be found below: ClinVar, SCV002054099.

## Ethics Statement

Ethical review and approval was not required for the study on human participants in accordance with the local legislation and institutional requirements. Written informed consent to participate in this study was provided by the participants' legal guardian/next of kin.

## Author Contributions

TX, KG, YL, YY, JL, ZT, and LX contributed to conception and design of the study. TX completed the experiment, performed the statistical analysis, and wrote the first draft of the manuscript. HG, LW, and RL wrote sections of the manuscript. All authors contributed to manuscript revision, reading, and approved the submitted version.

## Funding

This study was supported by the National Science Foundation for Young Scientists of China (8150020951) and the Natural Science Foundation for Young Scientists of Hunan Province (2016JJ4099).

## Conflict of Interest

The authors declare that the research was conducted in the absence of any commercial or financial relationships that could be construed as a potential conflict of interest.

## Publisher's Note

All claims expressed in this article are solely those of the authors and do not necessarily represent those of their affiliated organizations, or those of the publisher, the editors and the reviewers. Any product that may be evaluated in this article, or claim that may be made by its manufacturer, is not guaranteed or endorsed by the publisher.

## Supplementary Material

Supplementary Figure 1 Sanger sequencing verified the plasmid (upper, WT; below, Mutant. The box shows the variant sites).

Supplementary Table 1 qPCR primers.
