# Supplementary figures and images for: Whole-Exome Sequencing Identifies a Novel Variant (c.1538T > C) of TNNI3K in Arrhythmogenic Right Ventricular Cardiomyopathy
Source: Front Cardiovasc Med. 2022 Feb 22;9:843837. doi: 10.3389/fcvm.2022.843837 (PMC8902045; doi:10.3389/fcvm.2022.843837)

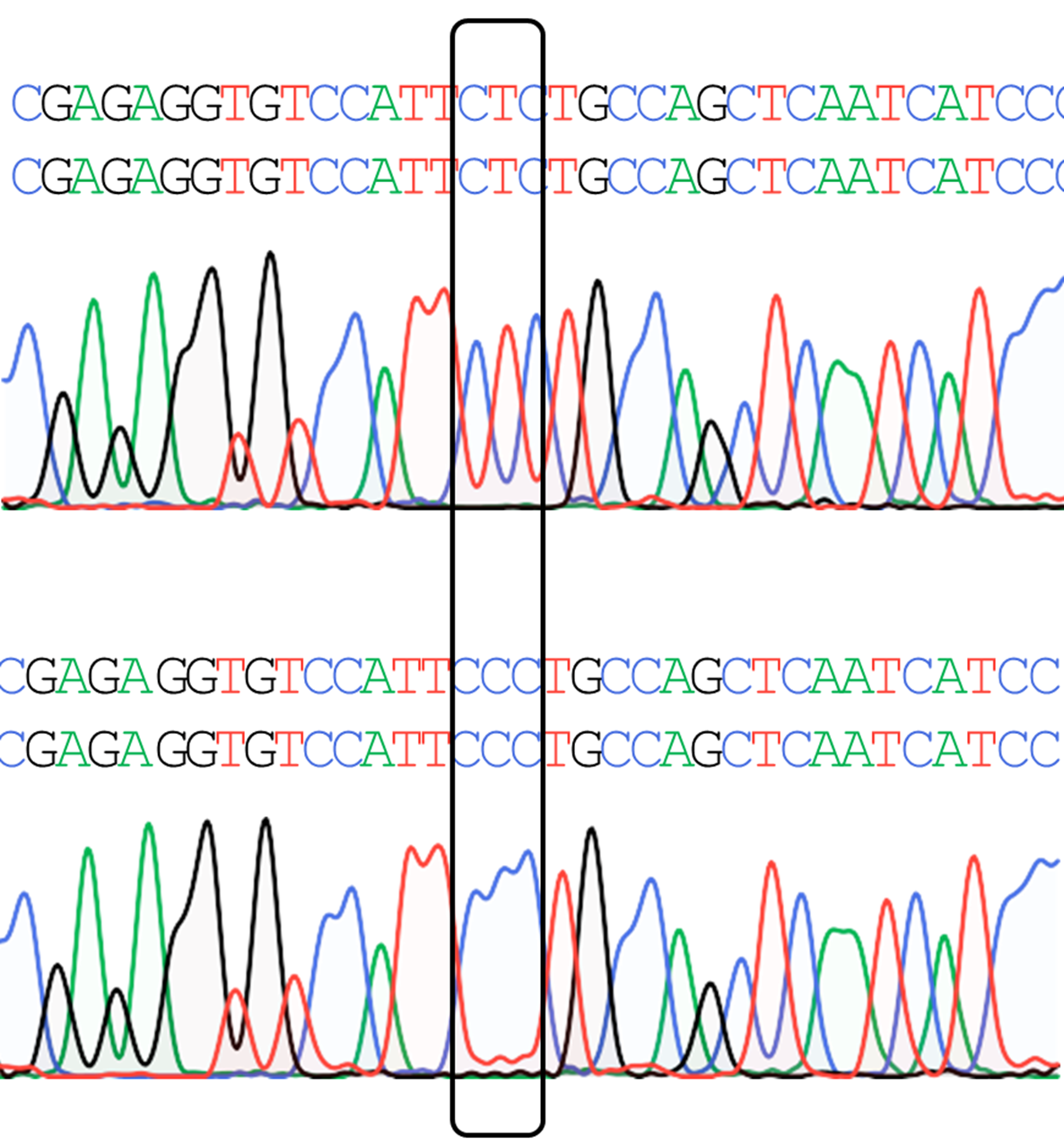

Supplement: Supplementary Figure 1 — Sanger sequencing verified the plasmid (upper, WT; below, Mutant. The box shows the variant sites). [file Image_1.TIF]
