# Supplementary material for: Whole-Exome Sequencing Identifies a Novel Variant (c.1538T > C) of TNNI3K in Arrhythmogenic Right Ventricular Cardiomyopathy
Source: Front Cardiovasc Med. 2022 Feb 22;9:843837. doi: 10.3389/fcvm.2022.843837 (PMC8902045; doi:10.3389/fcvm.2022.843837)
Supplement: Supplementary Table 1 — qPCR primers. [file Table_1.docx]

Supplementary Table 1. qPCR primers

| Gene name | Primer sequence (5’ 3’) |
| --- | --- |
| TNNI3K | CTATGCTCTGTGTCTGTGGG |
|  | TGATATGGGCTTGGGAATGG |
| RYR2 | AATGGACTGGAGATTGGCTG |
|  | CATTGGGACTTGTAGCTTGTG |
| GAPDH | AATCCCATCACCATCTTCCAG |
|  | AAATGAGCCCCAGCCTTC |
